# Annotations

**Published:** 1905-07-08

**Authors:** 


### ANNOTATIONS.

### The Birthday Honours.

THE medical profession is not strongly represented in the recent list of distinctions conferred by the King, but there will, we think, be general approval of the selection, and we heartily congratulate the fortunate recipients. Sir Thos. McCall Anderson is a graduate of the University of Glasgow, and occupies the chair of medicine in his alma mater. He is well known as an accomplished and successful physician, who, whilst having a wide sympathy with general medicine, has cultivated more particularly the department of dermatology. His recognition is a compliment to the great University in which he occupies a foremost place. Sir Thos. F. Chavasse is senior surgeon to the Birmingham General Hospital, and occupies a secure position in the professional and social life of the capital of the Midlands. His numerous writings on surgical subjects show him to be an experienced surgeon, and the value of his opinion is well recognised. In Sir Wm. J. Smyly the profession in Ireland has a distinguished representative whose work in the department of gynæcology has rendered his name familiar in many lands. His many friends and former pupils will heartily congratulate him on a well-deserved honour. Sir Philip Sydney Jones and Sir Edmond Sinclair Stevenson occupy distinguished positions in New South Wales and Cape Colony respectively, and in them our colonial brethren are fitly represented. Sir Chas. MacDonagh Cuffe, K.C.B., receives his promotion after many years of service in the Army Medical Department. Lieutenant-Colonel Wm. Coates, C.B., is the officer commanding the Manchester companies of the R.A.M.C. (Volunteers), and Dr. Wm. Thos. Prout, who receives the C.M.G., is principal medical officer in the colony of Sierra Leone. Cordial congratulations must certainly be offered to Surgeon-Major Thos. Egerton Hale, who, on the occasion of the jubilee of the Crimean War, in which he won the V.C., receives the distinction of a Companionship of the Bath. A similar honour falls to Surgeon-General W. C. Gubbins of the Indian Medical Service.

### Voyages d'Etudes Médicales.

WE have on previous occasions drawn the attention of our readers to these organised annual visits to the French health resorts, and as the tour for the present year has now been arranged, we have pleasure in again commending the scheme to the consideration of medical practitioners who may be discussing plans for the holidays. The "voyage" for 1905 is to commence at Luchon on Friday, September 1, and will end at Arachon on Thursday, September 14. It will include the health stations in the Western Pyrenees, and, amongst others, visits will be paid to such popular resorts as Pau, Biarritz, Cauterets, Eaux-Bonnes, and St. Sauveur. That in itself is an attractive programme, but, whilst the educational value of these tours to the practitioner is considerable, and every opportunity is afforded to those anxious to study the climatological and balneological value of the several stations, these advantages, it is to be remembered, are to be obtained under the most favourable auspices and at a very modest outlay. The experience of previous years has shown that all the arrangements are admirably planned and are carried out in a most successful manner. The party travels by first-class special train, and the hotels selected are beyond reproach. The various stations display an abundant hospitality to the visitors, and the social attractions of the tour are always considerable. English medical practitioners in increasing numbers are each year availing themselves of these tours, and are finding in them both a pleasant and a useful holiday. Regarding expense, the inclusive cost of the "voyage" is £12. This includes everything from the start at Luchon to the close of the trip at Arachon. In addition, the French railways grant a reduction to the extent of half the fare to those travelling either to the rendezvous or from the station where the tour closes. On the present occasion, as in former years, Professor Landouzy will be in command, and his genial and efficient direction is a guarantee of success. Full particulars can be obtained from Dr. Carron de la Carrière, 2 Rue Lincoln, Paris; or from Dr. Leonard Williams, 8 York Street, Portman Square, W.

### Hospital Staffs and Holidays.

POSSIBLY no order of work better deserves and needs an adequate holiday than that discharged by the medical practitioner. Unfortunately, however, no stay comes to the tale of sickness and disease, and hence, though the doctor may for a season cease from his labours, the patients remain and require attention. In private practice this may usually be managed by mutual arrangement between neighbouring practitioners or by securing the services of a *locum tenens.* But in hospital work the problem is not quite so simple. There are so many individuals to be considered and responsibilities of so wide an extent to be discharged. The time is brief, and all arrangements have to be fitted into a narrow compass. It is open to question whether these claims permit the work in our hospitals during the holiday season to be discharged in as satisfactory a manner as could be desired. Individuals do their best, but circumstances are sometimes too strong for them. A physician or surgeon saddled with two or three times the usual number of beds is obviously placed under unfair responsibilities, and patients in such circumstances can hardly receive a full measure of attention. It is, of course, well known that the absence of the seniors gives a chance to the junior members of the staffs to undertake the work of the wards, with advantage to themselves and by no means to the prejudice of the patients. But often a junior in this position is called upon to accept the work of more than one senior, and perhaps, in addition, has also to continue his attendance on out-patients. This seems to us a most unfair demand to make on the junior staff. The responsibility for all holiday arrangements should be accepted by the hospital managers, and after the members of the staff have adjusted their plans so as to secure the maximum attention for the hospital, the managers should appoint and pay a reasonable fee to temporary officers to fill up the gaps.

